# Genetic Variants Involved in Bipolar Disorder, a Rough Road Ahead

**DOI:** 10.2174/1745017901814010037

**Published:** 2018-02-28

**Authors:** Germano Orrù, Mauro Giovanni Carta

**Affiliations:** 1 Department of Surgical Sciences, Molecular Biology Service (MBS), University of Cagliari, Cagliari, Italy; 2 National Research Council of Italy, ISPA, Sassari, Italy; 3 Department of Medical Sciences and Public Health, University of Cagliari, Cagliari, Italy

**Keywords:** *Bipolar disorder*, *Genome-Wide Association Studies (GWAS)*, *Candidate genes*, *Polymorphisms*, *Molecular beacons*, *Mutations*

## Abstract

**Background:**

Bipolar Disorder (BD), along with depression and schizophrenia, is one of the most serious mental illnesses, and one of the top 20 causes of severe impairment in everyday life. Recent molecular studies, using both traditional approaches and new procedures such as Whole-Genome Sequencing (WGS), have suggested that genetic factors could significantly contribute to the development of BD, with heritability estimates of up to 85%. However, it is assumed that BD is a multigenic and multifactorial illness with environmental factors that strongly contribute to disease development/progression, which means that progress in genetic knowledge of BD might be difficult to interpret in clinical practice.

**Objective:**

The aim of this study is to provide a synthetic description of the main SNPs variants identified/confirmed by recent extensive WGS analysis as well as by reconstruction in an *in vitro* mechanism or by amygdala activation protocol *in vivo*.

**Method:**

Bibliographic data, genomic and protein Data Banks were consulted so as to carry out a cross genomic study for mutations, SNPs and chromosomal alterations described in these studies in BD patients.

**Results:**

Fifty-five different mutations have been described in 30 research papers by different genetic analyses including recent WGS analysis. Many of these studies have led to the discovery of the most probable susceptibility genes for BD, including ANK3, CACNA1C, NCAN, ODZ4, SYNE1, and TRANK1. Exploration has started the role of several of these mutations in BD pathophysiology using *in vitro* and animal models.

**Conclusion:**

Although new genomic research technology in BD opens up new possibilities, the current results for common variants are still controversial because of four broad conditions: analytical validity, clinical validity, clinical utility and a reasonable cost for genetic analysis are not yet accessible.

## 1 INTRODUCTION

Bipolar disorder is a complex and multifactorial illness associated with a form of human Mood Disorders (MD) and, along with depression and Schizophrenia, it is one of the most serious mental diseases. Different epidemiological 

researches have reported that the estimated lifetime prevalence of bipolar disorder among adults worldwide is 1 to 3 percent for at least of a series of mental disorders in the last years and it is estimated that at least 60 million people will be affected [1]. Furthermore, this information is incomplete and underestimates the problem, because only a small number of disorders are included and no data is reported on the high risk population, especially the over 65s [2, 3]. According to the International Classification of Diseases (ICD) MD falls into two main groups: (i) elevated mood i.e. mania, hypomania or depressed mood, also known as major depressive disorders, or (ii) moods represented with cycled steps of mania and depression such as Bipolar Disorders (BD) [4]. For these mental symptoms, different studies on family and twins explicitly demonstrate a solid contribution of inherited genetic factors to the risk of some MDs [5], even though traditional linkage procedures have yet to identify any gene able to increase the risk of MD, for example by a Mendelian mechanism. The clinical diagnosis for MD is made on the basis of a combination of certain clinical indicators based on a list of diagnosis criteria. However, the individual symptoms are not specific and may vary considerably from person to person and over the disease course. Furthermore, the symptoms of BD are common to many psychiatric disorders and this aspect makes a clinical diagnosis for MD even more difficult. In fact, diagnostic boundaries are not clearly defined, and not uncommonly, patients change diagnoses over the course of a lifetime [6]. At present, MD and also BD have resulted as deriving from multifactorial genic and epigenetic factors when the weight of the genetic counterparts resulted as the sum of different Single Nucleotide Polymorphisms (SNPs) [7, 8]. An enormous technical contribution in explaining the genetics of MD has recently come from Genome-Wide Association Studies (GWAS) and whole genome sequencing obtained by Next Generation Sequencing procedures (NGS) [9, 10]. This molecular approach has led to renewed interest for rarer polymorphic forms of genetic variation in complex non-mendelian phenotypes such as: psychiatric illnesses [8, 9] or intellectual disability. This technology provides an extensive investigation of genomes, and the obtained results could thus be promising and readily applicable to the diagnosis of BD associated genetic features. Moreover, the post-analyses interpretation of NGS native data remains difficult, due to the size and complexity of the human genome and to technical errors in pre-analytical and post analytical steps and also because most mutations related to BD are not located in genes and have an unknown biological mechanism. This is why additional experiments are needed to codify a specific mutation, for example, the GWS data needs to be crossed with transcriptome and proteome data such as non-coding ribonucleic acid and MicroRnas profiles [11, 12]. Thus, the informed use of reference standards, and associated statistical principles ensures a rigorous analysis of NGS data which is essential in its future clinical use for MD laboratory diagnosis. However, this different approach has allowed Single Nucleotide Polymorphisms (SNPs) located in no coding regions to be investigated as possible candidates in these illnesses. The current strategy to confirm a statistically effective role in these illness is to revise the hot spot genic regions by less expensive and validated molecular procedures such as (i) Sanger or Pyrosequencing procedures and (ii) real time PCR base methods [13-15]. In this work, we describe genes and their nucleotide variants as the best candidates occurring in MD.

## 2 METHODOLOGY

This study was conducted using the PubMed database on articles published before November 17, 2017 and after 2000. Separate searches were made for genes, SNPs and Mutations by using: Uniport, SNPedia DisGenet and Psychiatric Genomics Consortium (PGC).

## 3 GENETIC OF BIPOLAR DISORDER

Different studies on twins, based on concordance rates, have suggested that BD shows a high heritability grade estimated to be between 60% and 85% [8, 16]. In the recent years, it has been linked to different biological processes, and to a certain extent, these mechanisms are controlled by a variety of genetic factors, some of which are not yet well-known. These genic candidates code for receptors, enzymes, transporters *etc*. and some of these have a role in disease occurrence/ progression and in drug response. Such variants can be relevant for the effects of the drugs that act on these structures. At the same time, it is important to emphasize that the effects of most of the medications used in psychiatry are not very specific and it is complicated to identify a set of mutations that can be used in clinical and drug therapy prediction for these diseases. As described before, genetic risk factors must be associated with epigenetic risk factors, such as patient habits, for example, alcohol dependence, abuse, *etc*. However, these factors often vary from person to person and are not easy to measure and quantify, bringing most clinicians to agree that a positive family history of bipolar disorder is actually not very common in everyday clinical practice [17]. In fact, if we perform a cross-sectional study between recent publications and search tools investigating human genetics such as the DNA databank SNPedia, we can observe a set of different mutations or SNPs that are associated to BD, even though most of the SNPs described as a BD risk factor were shown to be of low effect individually. Moreover, few are seen to be replicated in diverse populations as happen with rare variants such as chromosomal alterations, smaller insertions and deletions as well as Copy Number Variants, (CNVs) Fig. (**1**). This large amount of data must necessarily be considered in comparison to their role in disease onset, its progression and drug therapy failure, but most of the SNPs reported have been described using partial experimental data with a low number of analyzed subjects or an incomplete follow-up in a population study. In addition, for the majority of SNPs evidence is still lacking in animal models and **in vitro** by neuronal cell culture.

## 4 MAIN GENES AND MUTATIONS DESCRIBED IN BIPOLAR DISORDERS

Since the first Genome-Wide Association Study (GWAS) was performed for BD about ten years ago, a handful of risk loci have been identified which replicated in adequately sized follow-up studies with very large groups of patients and controls. In addition, for some of these genetic variations, additional experiments with animal models and neuronal cell cultures or using functional and structural brain imaging have been performed [18-22]. These different *in vivo*/*in vitro* approaches have resulted in findings in ANK3, CACNA1C, NCAN, ODZ4,, SYNE1 TRANK1, described here as the top candidate genes in BD risk [21], Fig. (**1**), Table (**1**).

### 4.1 ANK3

The protein Ankyrin-G is required in the clustering of sodium voltage gated channels in nodes of Ranvier and axonal segment and is involved in different biological functions as well as in maintenance of membrane domains. This modular protein resulted as being an essential factor for enabling the propagation of action potentials in myelinated neurons [23]. The allele C in rs10994415 resulted as being associated with BD which showed an odds ratio equal to 1.27 in WGS studies [24], Table (**1**).

### 4.2 CACNA1C

This gene codes for the alpha-1 subunit of a voltage-dependent calcium channel and is a transmembrane protein involved in a calcium channel. This channel represents an important neuronal regulator in different tissues, for example, as a neuronal regulator of muscular tissue contraction in the heart and skeleton. This protein could be involved in axon guidance and in synaptic transmission in the brain; in fact, some missense mutations in the gene coding region are responsible for Timothy syndrome in humans, in particular, rs1006737 and rs2159100 are the most studied SNPs variations in mood disorders [25, 26], Table (**1**). However, these SNPs are vulnerable to the confounding effects of ethnic admixture in genome-wide association studies. For example, the (G: G) variant of SNP rs1006737 reported in a Caucasian population could not be replicated with genome-wide significance in individuals of African descent or in some studies in Europe and Asia.

### 4.3 ODZ4

The hot spot mutation described for BD (and other psychiatric disorders) are the variants rs12576775 and rs17138171 positioned in the intronic gene region. This gene codifies for a transmembrane signal transduction protein (tendering protein 4). In European populations, the minor rs12576775 allele occurs in about 20% of individuals. It also appears to play a central role in the regulation of neuronal and synaptic connectivity during brain development. However, knowledge about ODZ4 is still very limited, and more functional studies are clearly needed before clinical applications could be considered [24, 26, 27].

### 4.4 NCAN

The overall frequency of the A allele of SNP rs1064395 is about 23%, reported in 51% of African populations and 15% of European subjects (odds ratio 1.17) [28]. The gene is located in chromosome 19 and codifies for a secreted protein located in the extracellular space in the Golgi apparatus and in the lysosomal cavities. This protein modulates cell adhesion, cell migration, and axon guidance.

### 4.5 TRANK1

This gene, also called LBA1, encodes for a TPR and ankyrin repeat-containing protein, and was originally identified as a brain-specific antigen in a murine model of systemic lupus erythematosus, a human disorder with frequent neuropsychiatric symptoms [29]. It has shown to be responsive to valproic acid in humans. The main studied SNP is rs9834970 which is located about 12 kb distal to the 3' UTR of TRANK1 (hg18). This variant resulted significant at the P = 2.4 × 10^−11^ level, with no heterogeneity [24].

### 4.6 SYNE1

The SNP rs9371601 described in the intergenic gene region is associated to BP with an odds ratio of 1.14 The gene encodes for Nesprin-1^α^, an outer nuclear membrane intracellular protein, which connects the nucleus to the cytoskeleton via its N-terminal region. Experimental data suggests that this protein and other related ones in the same family are well suited to orchestrate signaling between cell membranes and the cytoskeleton [30]. They are implicated in diseases such as cancer, myopathies, arthrogryposis, neurological disorders and hearing loss.

## 5 OTHER GENIC CANDIDATES

### 5.1 ADCY2

*ADCY2* encodes for an integral membrane signaling enzyme (Adenylate cyclase) involved in the production of cAMP that activates the Protein Kinase A (PKA) pathways that lead to changes in cellular metabolism and gene transcription patterns. In the brain this enzyme is activated downstream of the G Protein Coupled Receptors (GPCRs) implicated in neuropsychiatric disorders, and in this context, it is a signal integrator of GPCR signaling in the brain. The SNPs rs13166360 located in an exonic region are associated in the literature with BD [24, 31]. Fig. (**1**).

### 5.2 GSK3B gene

This gene encoded for a glycogen synthase kinase 3 beta, regulates gene expression for three fundamental biological processes: (i) cell adhesion, (ii) cell polarity and (iii) signal pathways (NF-RB, Hedgehog Wnt/b-catenin). Its putative role in mental disorder has been described [32], and some articles also focus on the effects of GSK3B polymorphisms on BD. Copy Number Polymorphisms (CNPs) could be associated with BD, whereas for SNP -50C/T (rs334558), associated in the past to BD, the latest report demonstrates a lack of association for this mutation with the risk for this illness [33].

### 5.3 CUX2 and FAM109A

The role of two SNPs (rs3847953 and rs933399) positioned in the chromosome 12 on the genes CUX2 and FAM109 respectively has been described by different studies [34, 35] Fig. (**1**).

## 6 DISCUSSION

Despite the “fog” due to the complex interpretation of genetic data by NGS analysis of complete genomes and their subsequent modulation with clinical data in a large variety of genetically different populations, this research could prove more promising than expected.

These extensive data, crossed with the clinical parameters for BD, report a set of possible candidates linked to six different genes, some of which have also been investigated by amygdala activity studies or **in vitro** models reporting a possible physiopathological role of these variants and bipolar disorder. The majority of these SNPs are gene variants located in no-coding regions, such as in the intronic gene section. This could require the study of the folding primary RNA transcript and its role in the folding process during the post-transcriptional process, for example, this approach is now used to optimize the DNA sequencing protocol in long human genes [36]. Another shortcoming of these procedures is that a transcriptome control could be useful for these studies, in particular for SNPs located upstream of the promoter region [37, 38]. However, the risk variants found so far shown too little variance to allow data to be used for individual prediction of the disease risk, course, or drug effect, in addition to the polygenic nature of the disease. Here, the effects of a large number of risk variants can be cumulatively recorded using new methods and can be integrated into polygenic risk scores that represent a higher proportion of the phenotypic variance.

Another problem is that NGS-GWS analysis is very expensive for many research groups. In fact, these studies involve big projects employing a large number of clinical and laboratory personnel. In this context, it could be useful to perform a “second research step” by using traditional molecular protocols, such as real time PCR probe array or Pyrosequencing procedures, which are currently successfully employed for human and microbial mutations [13-15, 39-46]. This aspect could allow field research to investigate the primary gene-SNPs reported with GWAS studies.

### 6.1 Usefulness of Molecular Screening in “Sub-Threshold” Mania Elements

A recent line of research has found that the possession of “sub-threshold” mania elements as an element not necessarily linked to a pathological condition [47]. For example, being immigrants from rural areas into the megacity of South America is associated with a greater positivity for sub-threshold mania than the population of origin, although without increased frequency of mood disorders [48, 49].

From this point of view, it would be very interesting to have an easy to use genetic screener in order to be able to verify whether possessing genetic variants considered “at risk” may under certain conditions express not pathology but adaptive potential. In this framework, it could be interesting to have an easy and inexpensive molecular technique thus available for large researcher groups.

In these last years, we have used a molecular procedure to detect different DNA sequences by using a high specify fluorescent molecular probes called Molecular Beacon (MB) [13, 14, 50, 51] and we thought that this procedure could be useful for a large molecular screening for SNPs variants in sub-threshold mania subjects. MB is a circular oligonucleotide characterized by a high specificity for the chosen SNP target and by its ability to develop a fluorescent forming reaction when used in a conventional PCR test [52]. With this procedure it is idealizable to perform a molecular platform able to reveal in the same time the top SNPs described previously with GWAS studies.

## CONCLUSION

This second study level could significantly increase the cases where clinical-molecular studies could be carried out in poor areas of the world. For example, there are currently no biological markers for possible different subgroups of BD. In the future, if greater number of disease-associated variants come to light along with their respective biological functions, and different patterns of risk variants can be demonstrated in patients with different etiologies, this would allow an etiologically justified differentiation of BD subtypes as well as sub-threshold subjects. Another important field of research that could not be presented here is epigenetics, an aspect that is still uncertain in the road of clinical governance of Bipolar disorder.

## FOOTNOTES

- https://www.snpedia.com/index.php/Bipolar_disorder
- Psychiatric Genomics Consortium (PGC): http://www.med.unc.edu/pgc
- http://projects.tcag.ca/variation/
- WHO report 2017: http://www.who.int/mediacentre/factsheets/fs396/en/
- UniProt data bank: http://www.uniprot.org/
- DisGenet: http://www.disgenet.org/web/DisGeNET/menu/search?1

## Figures and Tables

**Fig. (1):**
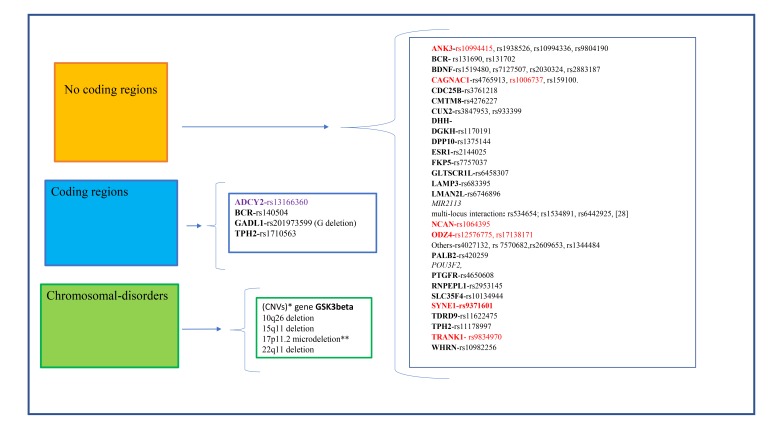
Schematic representation of the mutations associated to BD described in SNPedia and in bibliographic data.

**Table 1:** Top genetic variants associated with BD.

**Locus**	**Gene Name**	**SNP**	**Risk Allele**	**Odds Ratio**	**Reference**
10q21.2	ANK3	rs10994415	C	1.27	[24]
12p13.33	*CACNA1C*	rs1006737	A	1.14	[26, 27]
		rs2159100	T	1.18	
11q14	*ODZ4*	rs12576775	A	0.85-1.10	[24, 26, 27]
		rs17138171	C		
19p13.11	*NCAN*	rs1064395	A	1.17	[26, 28]
6q25	*SYNE1*	rs9371601	T	1.14	[26]
3p22	*TRANK1*	rs9834970	C	1.18	[53]
